# Detection and Characterisation of Circulating Tumour Cell Clusters in Neuroblastoma

**DOI:** 10.3390/cancers18030478

**Published:** 2026-01-31

**Authors:** Zoe Bell, Swathi Merugu, David Jamieson, Deborah A. Tweddle, Marina Danilenko

**Affiliations:** 1 Wolfson Childhood Cancer Research Centre, Translational & Clinical Research Institute, Newcastle University Centre for Cancer, Herschel Building, Newcastle upon Tyne NE1 7RU, UK; z.bell@newcastle.ac.uk (Z.B.); merugus@mskcc.org (S.M.); deborah.tweddle@newcastle.ac.uk (D.A.T.); 2 Translational & Clinical Research Institute, Newcastle University Centre for Cancer, Paul O’Gorman Building, Newcastle upon Tyne NE2 4HH, UK; david.jamieson@newcastle.ac.uk; 3 Department of Paediatric Oncology, Great North Children’s Hospital, Newcastle upon Tyne NE1 4LP, UK

**Keywords:** circulating tumour cells, circulating tumour cell cluster, neuroblastoma, metastasis

## Abstract

**Background:** Neuroblastoma (NB) is the most common extracranial paediatric solid cancer, with a 50% survival rate for high-risk patients. Circulating tumour cells (CTCs) are malignant cells shed by the primary tumour and metastatic sites that circulate in the bloodstream. CTCs form clusters with themselves (homotypic) or other cell types (heterotypic). **Objectives:** To use previously generated ImageStreamX Imaging Flow Cytometer data from blood samples from 24 patients across all NB risk groups, to examine for the presence of CTC clusters. **Methods:** Immunofluorescence and brightfield morphology were used to identify clusters followed by analysis using IDEAS image analysis software. **Results:** The mean number of clusters detected per sample was 87 (range, 0–725). Of the clusters detected, 1967/2094 (93.9%) were heterotypic and only 127/2094 (6.1%) were homotypic and found in 6/24 patients. Interestingly, in 3/24 patients, at least one cluster (median, 2 and range, 1–18) was found, but no single CTCs were detected. Clusters mostly comprised two cells (62.8%), with the maximum number of cells in homotypic and heterotypic clusters being four and eight, respectively. **Conclusions**: These results highlight that imaging flow cytometry can be used to detect and characterise CTC clusters in peripheral blood samples from NB patients, leading to further research exploring the composition and role of CTC clusters in NB metastasis.

## Simple Summary

Neuroblastoma is an aggressive childhood cancer. During disease progression, neuroblastoma tumours can release circulating tumour cells (CTCs) into the bloodstream. These cells can group together to form clusters, which may give rise to distant metastases and are linked with worse prognosis in other cancer types. In this study, we used imaging flow cytometry to detect and characterise CTC clusters present in blood samples from neuroblastoma patients. Clusters were detected in 79% of patients. Most clusters (93.9%) contained tumour cells grouped with other cell types, while a smaller proportion (6.1%) consisted of cancer cells only. Each cluster contained between 2 and 8 cells. Our study highlights the importance of studying CTC clusters in neuroblastoma and supports further research into the role of clusters in metastatic spread.

## 1. Introduction

Neuroblastoma (NB) is the most common extracranial paediatric solid tumour, accounting for 8–10% of childhood cancers with a 50% survival rate for high-risk patients (defined as metastatic disease over the age of 1 year of *MYCN* amplified NB) [1]. Invasive tumour biopsies, currently performed to determine treatment for NB patients, are associated with high morbidity, especially in patients below 18 months old. Liquid biopsies, collected to assess circulating tumour cells (CTCs) and circulating tumour DNA (ctDNA), offer a safer alternative and can also detect intra-tumoral heterogeneity [2].

CTCs are malignant cells shed by the primary tumour or metastatic lesions and circulate in the bloodstream. They can form clusters, which promote survival and metastasis. Homotypic clusters comprise only tumour cells, while heterotypic clusters form with other cell types or cell fragments. CTC clusters have been detected in a range of cancer types including melanoma, lung cancer, and breast cancer [3]. CTC clusters have been shown to be more successful in forming distant metastases compared to single CTCs, resulting in a poorer prognosis in breast and prostate cancer [4].

Although single CTCs in NB have been reported [5,6,7], less is known about CTC clusters. A previous study focusing on NB CTC clusters in 61 patients at diagnosis used microfluidic chips for cluster isolation from peripheral blood and found that most of the detected clusters were homotypic [8].

CTC cluster analyses offer the potential to understand metastasis initiation in NB. The purpose of this study was to detect and characterise CTC clusters in a NB patient cohort involving patients from all risk groups, at diagnosis and relapse, using previously acquired ImageStreamX (ISx) Imaging Flow Cytometer data [5], and to compare findings to matched single CTC data where possible.

## 2. Results

### 2.1. Detection of CTC Clusters in the Absence of Single CTCs

CTCs and CTC clusters were detected in 16/24 (67%) and 19/24 (79%) patients, respectively (Table 1). Clusters were found in 2/4 low and 17/19 high-risk patients. Blood samples, collected at diagnosis and relapse, as well as those with and without bone marrow involvement, had CTC clusters. Interestingly, in three patients (4, 19, and 22), no single CTCs were identified, but at least one cluster was detected, while two other patients (6 and 14) had low numbers of single CTCs but higher numbers of clusters identified. In contrast, five other patients (7, 13, 15, 16, and 17), had low numbers of clusters but high single CTC counts.

### 2.2. Detection of Homotypic and Heterotypic NB CTC Clusters

**Figure 1:**
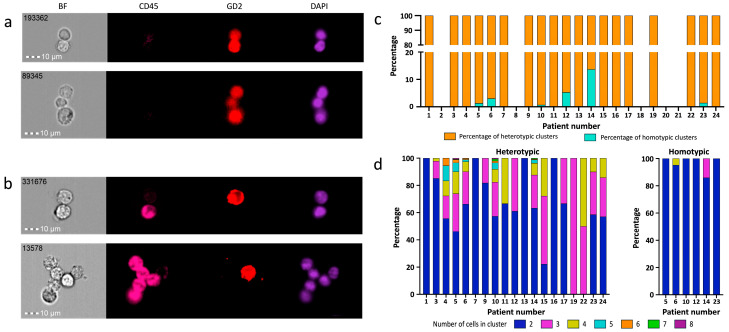
Examples of CTC clusters found in blood samples. (**a**) Immunofluorescence images of two homotypic clusters consisting of two and three NB cells (GD_2_+ve/CD45−ve/DAPI+ve). (**b**) Examples of heterotypic clusters consisting of one NB cell with one immune cell (GD_2_-ve/CD45+ve/DAPI+ve) and one NB cell with five immune cells. (**c**) Bar chart showing the percentage of homotypic and heterotypic CTC clusters for each patient. (**d**) Bar charts showing the percentage of clusters containing 2, 3, 4, 5, 6, 7, or 8 cells for heterotypic and homotypic clusters.

Both homotypic (Figure 1a) and heterotypic (Figure 1b) CTC clusters were detected. Of the total clusters detected, 1967/2094 (93.9%) were heterotypic and identified in all cases containing clusters (19/24 patients), and 127/2094 (6.1%) were homotypic and only found in 6/24 high-risk patients (5, 6, 10, 12, 14, and 23) (Figure 1c). Homotypic clusters only accounted for a maximum of 13.7% of the total clusters found in a sample (median number of homotypic clusters per sample = 2.5, range = 1–99). Spearman’s Rank correlation test showed that there was a positive correlation between the number of single CTCs and the number of heterotypic clusters (*p* = 0.002) and between the numbers of homotypic and heterotypic clusters (*p* < 0.001). No significant association was observed between the numbers of homotypic and heterotypic clusters and patient risk group (Fisher’s exact test, *p* > 0.999) or patient survival status (chi-square test, *p* = 0.160).

### 2.3. Characterisation of CTC Clusters

**Figure A1:**
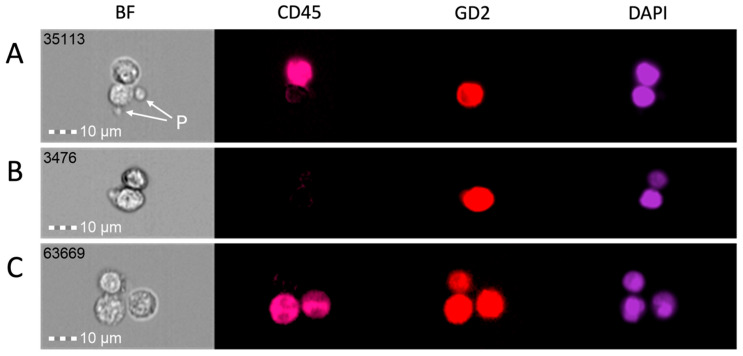
Immunofluorescence images showing examples of CTC clusters found in patient blood samples. (**A**) Cluster containing one NB cell and one immune cell with cell fragments that are possibly platelets, annotated in the image as P. (**B**) Cluster containing one NB cell with a GD_2_-ve/CD45−ve/DAPI+ve cell. (**C**) Cluster containing one NB cell with two larger GD_2_+ve/CD45+ve/DAPI+ve cells.

CTC clusters comprised between 2 and 8 cells. The median cluster size comprised two cells (62.8% of all clusters, Appendix A.1, Table A1). In homotypic clusters, the maximum number of cells was four (mean and median = 2, range = 2–4), and it was eight in heterotypic clusters (mean and median = 2, range = 2–8) (Figure 1d). Some clusters contained unidentified cells or cell fragments (Appendix A.2, Figure A1), with further testing required to identity these.

## 3. Discussion

Compared to single CTCs, CTC clusters have been reported to be up to 100 times more effective in establishing metastases [4]. Their assessment in liquid biopsies might therefore provide a valuable insight into the metastatic potential of cancer.

The results of this study show that imaging flow cytometry can be used to detect and characterise both homotypic and heterotypic CTC clusters in NB. Interestingly, 21% of patients had no/low counts of single CTCs but high numbers of CTC clusters. This finding highlights the importance of examining liquid biopsies for the presence of CTC clusters in NB to gain further insights into disease biology.

Most CTC clusters identified in this study were heterotypic and were detected in 19/24 patients (79%), with only 6/24 patients (25%) showing homotypic clusters. The only previous study characterising NB CTC clusters reported that clusters were homotypic in 61/64 patients (95.3%) and heterotypic in only 3/64 patients (4.7%) [8]. However, studies on breast cancer [9], lung cancer [10], and glioma [11] all reported a higher frequency of heterotypic clusters (detected in 75.49%, 29.27%, and 33.3% of patients, respectively). The possible explanation for discrepancies between the current and the previous NB CTC cluster studies could be linked to the different methods applied for cluster detection (imaging flow cytometry and microfluidic chips, respectively). Thus, we propose that CTC sample processing is refined to preserve both cluster types during isolation from patient blood samples, carefully optimising immune cell depletion and cluster enrichment steps.

To our knowledge, this is the first study of NB CTC clusters to report cluster sizes. Interestingly, most of the detected CTC clusters consisted of a pair of cells, but clusters comprising eight cells were also observed. To suppress the metastatic potential of CTC clusters, treatments are being developed to reduce their size. A proof of concept clinical trial using Digoxin, a Na+/K+ ATPase inhibitor, was carried out in patients with breast cancer and showed that Digoxin was able to reduce the size of both homotypic and heterotypic clusters [12]. The ability to detect and break down large CTC clusters is important, because larger clusters are likely to be more aggressive and resistant to environmental changes [13].

Additional cell types (e.g., possible platelets and cancer-associated fibroblasts) were also concurrently observed in NB clusters (Appendix A.2, Figure A1A,B). Further testing is required to confirm the identity of these cell types. Some cells in the clusters were GD_2_+ve/CD45+ve/DAPI+ve (Appendix A.2, Figure A1C) and could be tumour cells engulfed by macrophages, as previously observed in NB bone marrow samples [14].

In summary, our study highlights the potential importance of CTC clusters in NB for triggering metastases, which may help to identify potential new treatments preventing metastasis. However, this study is limited by its small size. Furthermore, GD_2_ expression may become downregulated in neuroblastoma patients following chemotherapy or immunotherapy, potentially compromising CTC and CTC cluster detection in cases with a weak or absent GD_2_ signal. A future study is needed, both to validate the current results and to enable survival analyses.

## 4. Materials and Methods

### 4.1. Patient Samples

Blood samples (1.5–10 mL) were collected from patients with NB from five UK Paediatric Oncology Principal Treatment Centres following institutional review board approval (ethics reference number 14/NW/0154), local institutional approval, and written informed consent from parents or guardians [5]. This study was undertaken in accordance with the ethical principles of the Declaration of Helsinki. Samples were collected in Cell Save (Veridex, Menarine Diagnostics, Florence, Italy) tubes, sent by post at room temperature within 72 h of collection, and processed within 96 h of collection. Prior to analysis on the ISx, blood samples were subjected to CD45 depletion, fixation, and cell membrane permeabilization. A healthy volunteer blood sample and DMSO (dimethyl sulfoxide) samples (Sigma-Aldrich, Dorset, UK, D2650) were used as negative controls in flow cytometry assays. Human neuroblastoma cell lines (SHSY5Y) spiked into healthy volunteer blood samples served as positive controls in the flow cytometry experiments. To visualise the cells during imaging flow cytometry, cells were stained with immunofluorescent antibodies for GD_2_-PerCp (BD Pharmingen, San Diego, CA, USA, 563438, 14.G2a) and CD45- PE-Cy 7 (BioLegend, San Diego, CA, USA, 560915, H130), and nuclei were stained with DAPI [5]. Data obtained from blood samples from 24 patients were included in this study (Table 1).

### 4.2. Criteria for CTC Cluster Selection

NB cells from our previous study were identified as GD_2_+ve/CD45−ve/DAPI+ve cells and immune cells as GD_2_-ve/CD45+ve/DAPI+ve. Other, non-NB cells were identified as GD_2_-ve/CD45−ve/DAPI+ve or GD_2_+ve/CD45+ve/DAPI+ve cells. A cluster was defined by the presence of at least one NB cell in contact with at least one additional DAPI+ve cell. For inclusion in this study, clusters comprised intact cells confirmed using brightfield (BF) cellular morphology images. Homotypic clusters were defined as at least two NB cells, whereas heterotypic clusters were defined as at least one NB cell with at least one other cell.

### 4.3. Identification of Circulating Tumour Cell Clusters and Cluster Analysis

ISx data analysis files (.daf) from our previous study [5] were analysed for CTC clusters using IDEAS image analysis software (Amnis IDEAS 6.4). During data acquisition, several files were created for each sample to mitigate data processing issues arising from one large file.

To detect clusters, a histogram of object area was created and gated to obtain a population of objects that were larger than a single cell. This population was then arranged from high to low GD_2_ expression, and each object was visually assessed for the inclusion criteria. Clusters were identified based on BF morphology and fluorescence expression, tagged, and manually counted. The percentage of clusters containing different numbers of cells, as well as proportions of homotypic or heterotypic clusters, was calculated. The numbers of CTC clusters were also compared to the numbers of single CTCs obtained previously [5].

### 4.4. Statistical Analysis

All statistical tests were performed using Prism 10.1.1 software; *p*-values were considered significant if *p* ≤ 0.05.

## 5. Conclusions

This study demonstrates that imaging flow cytometry is a robust approach for detecting and characterising CTC clusters in peripheral blood samples from neuroblastoma patients. The predominance of heterotypic clusters, alongside fewer homotypic clusters, highlights the complexity and heterogeneity of neuroblastoma liquid biopsies. These findings prompt further investigation into the biological and clinical significance of CTC clusters in neuroblastoma, particularly their potential contribution to metastatic dissemination.

## Figures and Tables

**Table 1:** CTC clusters detected by ImageStream Imaging Flow Cytometer (ISx). Table includes information about the patient cohort [5], numbers of CTC clusters and previously detected single CTCs [5]. Abbreviations: DOD, died of disease; ADF, alive disease-free; TD, toxic death.

Patient Number	Diagnosis/ Relapse	Risk Group	Patient Status	Bone Marrow Involvement	Ploidy Status of CTCs	Sample Volume (mL)	Single CTCs/ Sample	Numbers of CTC Clusters/ Sample	Single CTCs/ mL	CTC Clusters/ mL
1	Diagnosis	Low	TD	No	Diploid	8.2	24	1	2.9	0.1
2	Diagnosis	Low	ADF	No	-	3.0	0	0	0.0	0.0
3	Diagnosis	High	DOD	Yes	Hyperdiploid	4.0	654	47	163.5	11.8
4	Diagnosis	High	ADF	Yes	Hyperdiploid	6.0	0	18	0.0	3.0
5	Diagnosis	High	ADF	Yes	Diploid	7.8	88	307	11.3	39.4
6	Diagnosis	High	ADF	No	Hyperdiploid	8.0	48	708	6.0	88.6
7	Diagnosis	High	ADF	No	Diploid	7.4	72	1	9.7	0.1
8	Diagnosis	High	ADF	Yes	Diploid	1.6	0	0	0.0	0.0
9	Diagnosis	High	DOD	Yes	Diploid	5.0	29	11	5.8	2.2
10	Diagnosis	High	ADF	Yes	Diploid	5.0	389	149	77.8	29.8
11	Diagnosis	High	ADF	Yes	Diploid	4.1	28	3	6.8	0.7
12	Diagnosis	High	DOD	Yes	Hyperdiploid	4.0	522	19	130.5	4.8
13	Diagnosis	High	DOD	Yes	Diploid	2.2	29	2	13.2	0.9
14	Diagnosis	High	DOD	Yes	Diploid	8.0	46	725	5.8	90.6
15	Diagnosis	High	ADF	Yes	Diploid	10.0	145	18	14.5	1.8
16	Diagnosis	High	ADF	Yes	Diploid	3.4	60	1	17.6	0.3
17	Diagnosis	High	ADF	Yes	Diploid	4.5	78	3	17.3	0.7
18	Diagnosis	High	DOD	Yes	-	7.0	0	0	0.0	0.0
19	Relapse	Low	ADF	No	-	1.5	0	1	0.0	0.7
20	Relapse	Low	DOD	No	-	6.0	0	0	0.0	0.0
21	Relapse	Intermediate	ADF	No	-	8.5	0	0	0.0	0.0
22	Relapse	High	DOD	No	Hyperdiploid	6.0	0	2	0.0	0.3
23	Relapse	High	DOD	No	Hyperdiploid	7.8	304	71	39.0	9.1
24	Relapse	High	DOD	Yes	Diploid	3.0	92	7	30.7	2.3

## Data Availability

The raw data files used in this manuscript are available on reasonable request by writing to the corresponding author.

## Abbreviations

The following abbreviations are used in this manuscript:

CTC	Circulating tumour cell
ctDNA	Circulating tumour DNA
ISx	ImageStreamX
NB	Neuroblastoma
DOD	Died of disease
ADF	Alive disease-free
TD	Toxic Death

## Appendix A

### Appendix A.1

**Table A1:** Number of cells per cluster for each patient sample (mean and median number of cells per cluster = 2 and range = 2–8).

Patient Number	Number of Cells Per Cluster						
	2	3	4	5	6	7	8
1	1	0	0	0	0	0	0
2	0	0	0	0	0	0	0
3	40	6	1	0	0	0	0
4	10	3	2	2	1	0	0
5	144	84	49	20	7	1	2
6	475	164	52	10	6	0	1
7	1	0	0	0	0	0	0
8	0	0	0	0	0	0	0
9	9	2	0	0	0	0	0
10	86	37	14	7	2	2	1
11	2	0	1	0	0	0	0
12	12	7	0	0	0	0	0
13	2	0	0	0	0	0	0
14	481	167	54	16	3	4	0
15	4	9	5	0	0	0	0
16	1	0	0	0	0	0	0
17	2	1	0	0	0	0	0
18	0	0	0	0	0	0	0
19	0	1	0	0	0	0	0
20	0	0	0	0	0	0	0
21	0	0	0	0	0	0	0
22	0	1	1	0	0	0	0
23	42	22	7	0	0	0	0
24	4	2	1	0	0	0	0
**Total**	1316	506	187	55	19	7	4

## References

[1] Nong J., Su C., Li C., Wang C., Li W., Li Y., Chen P., Li Y., Li Z., She X. (2025). Global, Regional, and National Epidemiology of Childhood Neuroblastoma (1990–2021): A Statistical Analysis of Incidence, Mortality, and DALYs. eClinicalMedicine.

[2] Singh J., Peters N.J., Avti P., Trehan A., Mahajan J.K., Menon P., Bansal D., Kanojia R.P. (2025). The Role of Liquid Biopsy in Neuroblastoma: A Scoping Review. J. Pediatr. Surg..

[3] Yang Y., Huang G., Lian J., Long C., Zhao B., Liu X., Zhang B., Ye W., Chen J., Du L. (2024). Circulating Tumour Cell Clusters: Isolation, Biological Significance and Therapeutic Implications. BMJ Oncol..

[4] Schuster E., Taftaf R., Reduzzi C., Albert M.K., Romero-Calvo I., Liu H. (2021). Better Together: Circulating Tumor Cell Clustering in Metastatic Cancer. Trends Cancer.

[5] Merugu S., Chen L., Gavens E., Gabra H., Brougham M., Makin G., Ng A., Murphy D., Gabriel A.S., Robinson M.L. (2020). Detection of Circulating and Disseminated Neuroblastoma Cells Using the ImageStream Flow Cytometer for Use as Predictive and Pharmacodynamic Biomarkers. Clin. Cancer Res. Off. J. Am. Assoc. Cancer Res..

[6] Tuo J., Zhao Z., Ma X., Liu Z., Yang B., Zhang M., He X. (2024). The Relationship between Circulating Tumor Cells in Peripheral Blood and Clinical Characteristics of Pediatric Neuroblastoma and Prognostic Evaluation. Pediatr. Hematol. Oncol..

[7] Wang Y., Cao N., Cui X., Liu Z., Yuan X., Chen S., Xu H., Yi M., Ti Y., Zheng F. (2024). Detection of Circulating Tumor Cells Using a Microfluidic Chip for Diagnostics and Therapeutic Prediction in Mediastinal Neuroblastoma. Eur. J. Pediatr..

[8] Xu H., Cao N., Dai W., Sima C., Yi M., Chen S., Chen X., Liu C., Yu U., Lang H. (2025). Circulating Tumor Cells and Clusters as Liquid Biomarkers for the Diagnosis and Prognosis of Neuroblastoma. BMC Cancer.

[9] Scholten D., El-Shennawy L., Jia Y., Zhang Y., Hyun E., Reduzzi C., Hoffmann A.D., Almubarak H.F., Tong F., Dashzeveg N.K. (2025). Double-Positive T Cells Form Heterotypic Clusters with Circulating Tumor Cells to Foster Cancer Metastasis. J. Clin. Investig..

[10] Li Z., Fan L., Wu Y., Niu Y., Zhang X., Wang B., Yao Y., Chen C., Qi N., Wang D.D. (2022). Analysis of the Prognostic Role and Biological Characteristics of Circulating Tumor Cell-Associated White Blood Cell Clusters in Non-Small Cell Lung Cancer. J. Thorac. Dis..

[11] Qi Y., Sun Q., Deng G., Zhang H., Xu Y., Li Y., Huang S., Li Y., Ye Z., Wang Y. (2021). Identifying Circulating Glioma Cells and Their Clusters as Diagnostic Markers by a Novel Detection Platform. Clin. Transl. Med..

[12] Kurzeder C., Nguyen-Sträuli B.D., Krol I., Ring A., Castro-Giner F., Nüesch M., Asawa S., Zhang Y.W., Budinjas S., Gvozdenovic A. (2025). Digoxin for Reduction of Circulating Tumor Cell Cluster Size in Metastatic Breast Cancer: A Proof-of-Concept Trial. Nat. Med..

[13] Campenni M., May A.N., Boddy A., Harris V., Nedelcu A.M. (2020). Agent-based Modelling Reveals Strategies to Reduce the Fitness and Metastatic Potential of Circulating Tumour Cell Clusters. Evol. Appl..

[14] Beiske K., Burchill S.A., Cheung I.Y., Hiyama E., Seeger R.C., Cohn S.L., Pearson A.D.J., Matthay K.K. (2009). Consensus Criteria for Sensitive Detection of Minimal Neuroblastoma Cells in Bone Marrow, Blood and Stem Cell Preparations by Immunocytology and QRT-PCR: Recommendations by the International Neuroblastoma Risk Group Task Force. Br. J. Cancer.

